# Combined pulsed laser deposition and non-contact atomic force microscopy system for studies of insulator metal oxide thin films

**DOI:** 10.3762/bjnano.9.63

**Published:** 2018-02-21

**Authors:** Daiki Katsube, Hayato Yamashita, Satoshi Abo, Masayuki Abe

**Affiliations:** 1 Graduate School of Engineering Science, Osaka University, 1-3 Machikaneyama, Toyonaka, Osaka 560-8531, Japan; 2 also at PRESTO, JST, 4-1-8, Honcho, Kawaguchi, Saitama. 332-0012, Japan

**Keywords:** atomic resolution, frequency modulation atomic force microscopy, insulator thin film, pulsed laser deposition

## Abstract

We have designed and developed a combined system of pulsed laser deposition (PLD) and non-contact atomic force microscopy (NC-AFM) for observations of insulator metal oxide surfaces. With this system, the long-period iterations of sputtering and annealing used in conventional methods for preparing a metal oxide film surface are not required. The performance of the combined system is demonstrated for the preparation and high-resolution NC-AFM imaging of atomically flat thin films of anatase TiO_2_(001) and LaAlO_3_(100).

## Introduction

The surfaces of metal oxides have attractive and useful properties, such as superconductivity, ferroelectricity and catalytic activity [1]. These properties are derived from the unique structure, the electron and spin interactions, the degree of freedom of the electron orbit and the lattice constant of the metal oxides. To understand and apply these characteristics, various studies have been conducted thus far, including investigations of physical properties by growth of ultra-high-quality metal oxide thin films [2–3], discovery of novel physical properties by fabricating heterointerfaces using different metal oxides [4–5], and expression of conductivity and magnetism by doping metal oxides with specific elements [6–7]. In studies of metal oxide surfaces at the atomic scale, developments of observation techniques have been advancing rapidly, especially in the field of transmission electron microscopy [5,8–13]. As atomic resolution methods, scanning probe microscopy including scanning tunneling microscopy (STM) [13–30] and non-contact atomic force microscopy (NC-AFM) [19,23,29,31–47] have played important roles. NC-AFM in particular can be used to elucidate the structures of surfaces at the atomic scale. An NC-AFM measures the shift in cantilever resonance due to the interaction force between the tip and the sample, hence it is possible to use it to observe any surface regardless of the conductivity of the material [19,23,31–47]. STM on the other hand can only be used for observations of insulators with a thickness of few atomic layers on conductive substrates [20–22].

For observations of surface atoms of metal oxides using NC-AFM and STM, it is critical to prepare atomically flat and clean surfaces. A standard method for obtaining clean surfaces of metal oxides is performing iterations of Ar^+^ sputtering and annealing at high temperatures [23–26,37,41–42]. Different conditions of annealing and sputtering result in different types of surface reconstruction even under the same preparation conditions [26]. As an example of an insulator metal oxide, an Al_2_O_3_(0001) surface prepared using iterations of annealing and sputtering was imaged with atomic resolution in recent studies [35,37]. Difficulties with this method are a long preparation time and a low reproducibility. Recently, connecting an STM with a pulsed laser deposition (PLD) system [13,27–28] or with molecular beam epitaxy [18] has increased the types of measurements possible. Such combined systems allow for in situ observations, from sample preparation to measurements. In order to image surface atoms of insulator metal oxides with atomic resolution, in this study, we have developed a combined system consisting of an NC-AFM and PLD operated in ultra-high vacuum (UHV) at room temperature. In situ sample preparation and measurements have made it possible to image surfaces of insulator metal oxides under conditions where the sample does not become contaminated. Furthermore, the sample preparation time is significantly reduced. In the “Experimental” section, the configuration of the combined NC-AFM/PLD system and the heating method for annealing insulator samples are described. Then, in the section “Results and Discussion”, we introduce a method for finding the appropriate conditions for obtaining atomically flat surfaces, which is critical for obtaining atomic resolution images stably with NC-AFM. Finally, we demonstrate atomic resolution NC-AFM imaging of anatase TiO_2_(001) and LaAlO_3_(100). Both materials are important in the field of materials science, and it has been challenging to form and image atomically flat and clean surfaces of these two oxides thus far.

## Experimental

**Figure 1:**
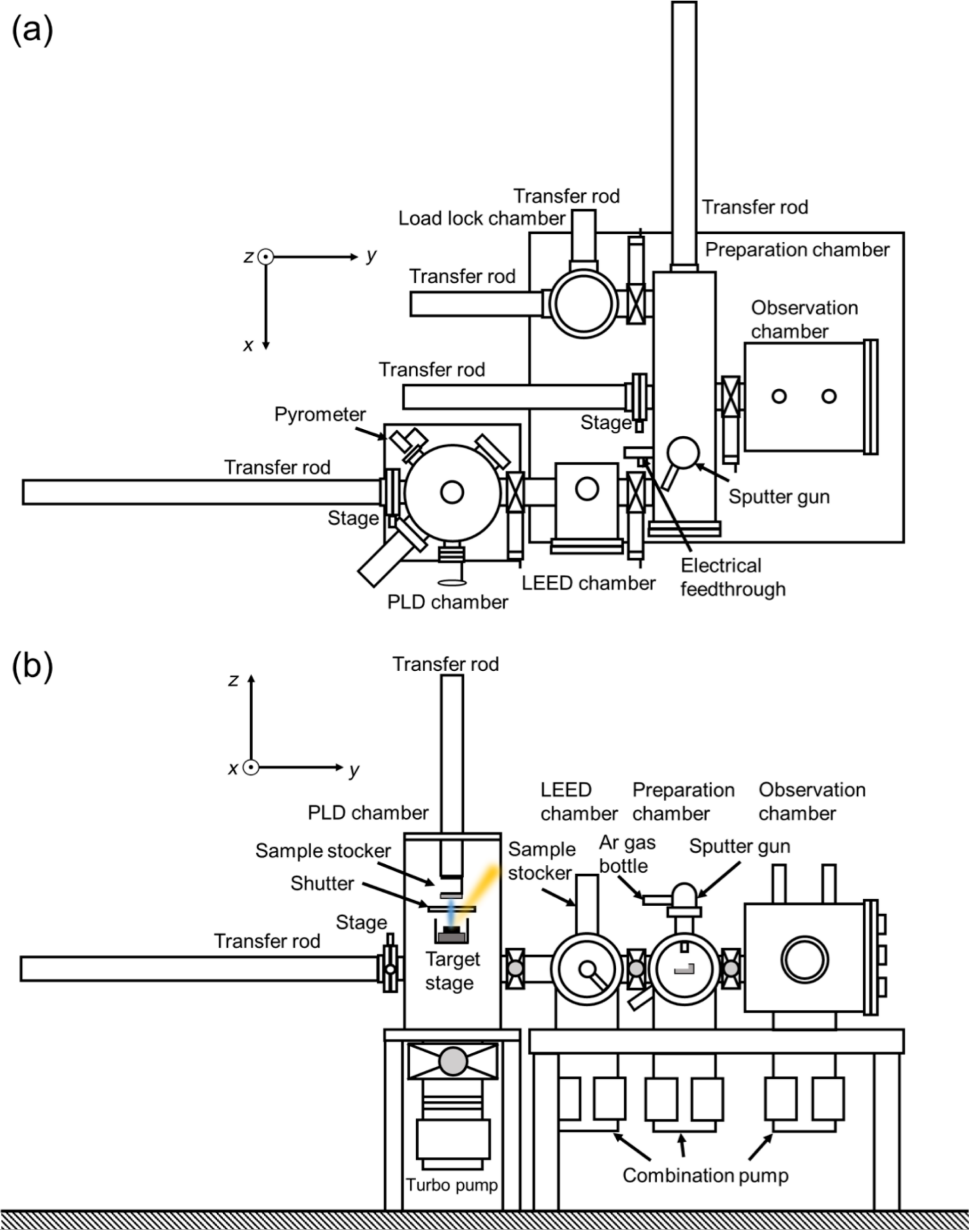
(a) Top view and (b) side view of the combined PLD/AFM system. STM measurements can also be performed. LEED and RHEED apparatuses are also installed. The AFM (and also STM) is operated at room temperature in UHV.

Figure 1 shows a schematic diagram of the combined NC-AFM and PLD system that was developed. It includes UHV chambers for sample preparation, low-energy electron diffraction (LEED), and load locking. It is also designed to allow for adding surface analysis capabilities such as reflection high-energy electron diffraction (RHEED). Sample preparation and NC-AFM, STM and LEED measurements can be performed while keeping the sample under UHV. The PLD chamber was designed to be as small as possible for easy sample exchange and transfer. In addition, a small design would lead to smaller vibrations during AFM and/or STM measurements and, thus, better observations. The PLD chamber the side of which is fitted with an CF203 port includes a PLD target, a shutter, a sample stocker, and a RHEED apparatus. A small size commercial PLD target system (UNISOKU) is installed in the PLD chamber. Piezoelectric motors are used to rotate the PLD targets. The pulse laser (COHERENT COMPex Pro; KrF, λ = 248 nm). The laser enters the chamber diagonally from above, so the sample surface faces downward during PLD sample preparation. In order to prevent the sample from falling, both the sample and the holder are placed on the stocker using magnets, and the sample and holder do not fall even when the temperature is over 1000 °C.

**Figure 2:**
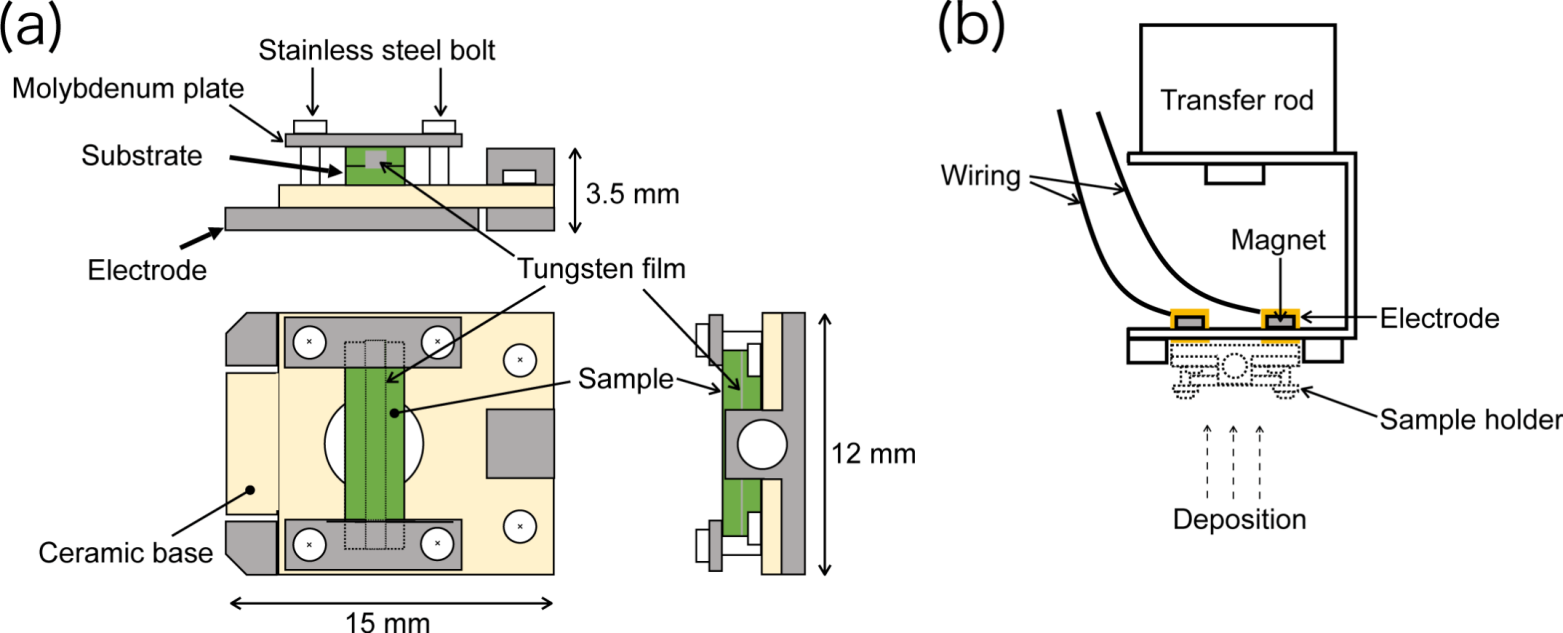
(a) Sample holder and (b) holder stocker used in the PLD camber.

During sample preparation with PLD, the sample is heated in an O_2_ atmosphere. If the sample is a doped metal oxide, it can be heated by flowing a current through the sample as done in previous STM and NC-AFM studies. To anneal insulator samples, a tungsten thin film is sandwiched between the sample and a metal oxide substrate [35,37]. The sample is then heated by flowing current through the tungsten film. The configuration of the sample and the sample holder used are shown in Figure 2a [43]. The substrate is fixed with four screws and leaf springs on the ceramic part of the sample holder. Stainless steel electrodes under the ceramic are used as connections for flowing a current through the tungsten thin film.

The electrode for the flowing current is insulated from the base of the stocker by insulator materials. This prevents a short-circuit of the flowing current to the UHV chambers. The U-shaped base plate of the sample holder stocker is 1 mm thick to reduce heat diffusion during heating of the substrate. The chambers of the NC-AFM, the sample preparation and the load lock are the same as in our previous studies [29,37,41,44–48]. To perform stable atomic resolution imaging in this chamber, a mechanism to fix the unit by double spring vibration isolation and eddy current damping [49] is provided, to prevent vibration noise. Therefore, it is possible to obtain atomic resolution images with the same performance as in our previous study [48]. We are able to perform NC-AFM measurements without the influence of vibration noise caused by the linkage to the PLD chamber. An optical interferometer [50] was used for deflection detection of the probe used for the NC-AFM observations. To image the insulator metal oxide thin films with atomic resolution, NC-AFM with the frequency modulation mode is used [51]. A commercial cantilever (Budget Sensors, TAP190) is used to obtain NC-AFM topographic images. An Ar^+^ sputtering gun is installed in the preparation chamber to clean the AFM tip apex as well as to clean the PLD-treated sample surface. Simultaneous AFM/STM [29] measurements can be performed using a conductive tip.

## Results and Discussion

### Preparation of atomically flat surfaces with PLD

For atomic scale observations of the metal oxide surface, it is necessary to prepare a clean and flat surface at the atomic level over a wide area. This is to avoid changes in the tip apex due to the presence of protrusions on the surface. Although there have been studies in which the growth of atomically flat metal oxides has been demonstrated [3,13,27–28,52–55], it is still difficult to find the best sample preparation conditions for PLD for atomic resolution imaging with NC-AFM and STM. Even if a clean diffraction pattern can be seen with RHEED performed during the PLD, it might be that the surface flatness necessary for the NC-AFM observation is not obtained. Parameters such as the sample temperature, oxygen partial pressure, and laser intensity during PLD need to be optimized for each sample. Since our UHV NC-AFM is optimized for atomic resolution measurements, scanning over a wide range cannot be performed. To find suitable conditions for efficient PLD deposition, a deposited sample was taken out into the ambient atmosphere and its topography was measured with a tapping AFM in air. Although it is difficult to obtain atomic resolution images in tapping mode, the larger scanning area of tapping AFM enabled us to check whether a step-and-terrace surface was formed over a wide range. Furthermore, the spatial resolution in the vertical direction of the tapping AFM is high, so images of a single-atomic step-and-terrace structure for confirming the flatness of the surface can be obtained. Evaluation using X-ray diffraction (XRD) in ambient air is also effective for checking the crystal structure, orientation plane, and crystallinity of a thin film. Once a single-atomic layer step is confirmed, a new sample is prepared under the same PLD conditions and in situ NC-AFM (and also STM) measurements can then be performed in UHV.

**Figure 3:**
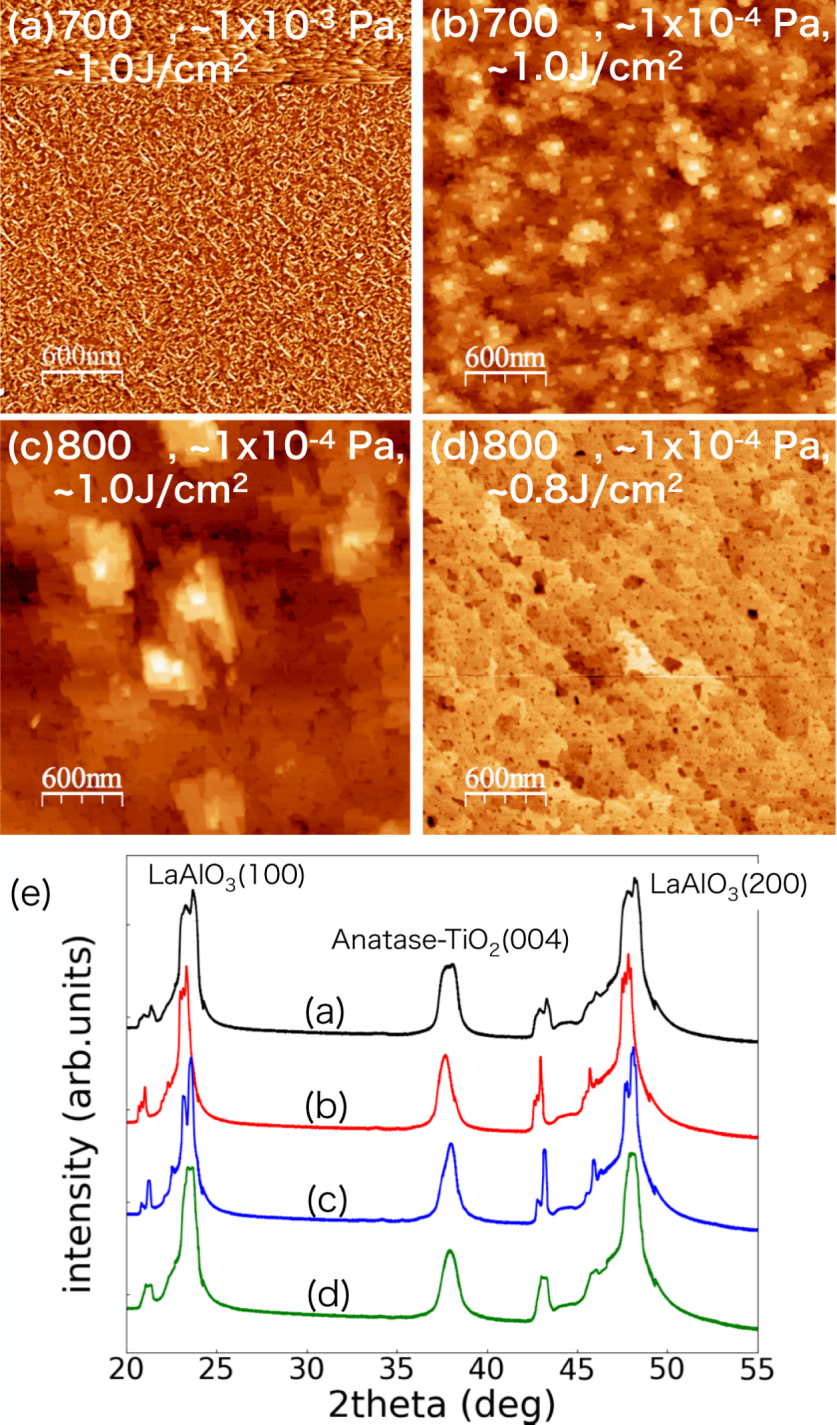
(a–d) Tapping mode AFM images of anatase-TiO_2_(001). (e) XRD patterns for the samples shown in (a–d). The samples were prepared under different conditions of annealing temperature, oxygen partial pressure, and laser power density as shown in the AFM images. The pulsed laser frequency was *f*_p_ = 2 Hz. The samples were washed with acetone and ultrapure water before performing PLD. AFM images are processed using the WSxM software [57].

As an example, a process flow for determining the sample preparation conditions of anatase TiO_2_(001) is shown in Figure 3 [56]. Referring to previous studies [52–53], we started the PLD with the following sample parameters: temperature *T*_s_ = 700 °C, oxygen partial pressure *P*_O_ ≈ 1 × 10^−3^ Pa, laser density *I* = 1.0 J/cm^2^, and laser repetition rate *f*_p_ = 2.0 Hz on a LaAlO_3_(100) substrate. The sample was then taken out of vacuum, and its surface was measured with tapping mode AFM in air as shown in Figure 3a. It was found that an atomically flat area did not exist on the surface, instead grain structures appeared on the surface. A peak for anatase TiO_2_(001) in the XRD pattern Figure 3e indicates that each small grain should contain anatase TiO_2_ crystals. Since excessive oxygen could cause the grain structure observed, the sample was prepared after reducing the oxygen partial pressure to *P*_O_ ≈ 1 × 10^−4^ Pa as shown in Figure 3b. In this case, many small islands including single- and multi-atomic steps were observed. In order to flatten the islands of the multi-atomic step structure, the sample temperature during the PLD was increased to *T*_s_ = 800 °C (Figure 3c). Increasing the annealing temperature then formed a flat area. Although anatase TiO_2_(001) is said to undergo a structural change to rutile TiO_2_(001) at around 750 °C, the XRD pattern showed that the anatase phase remained in this case. This is due to the lattice constant of the substrate. Considering that a phase transition from anatase to rutile may occur if the temperature is raised beyond 800 °C, we then changed the laser power density to a lower value (*I* = 0.8 J/cm^2^) to reduce the deposition rate (Figure 3d). Since we obtained a step-and-terrace surface, as shown in Figure 3d, we decided to use these conditions for the NC-AFM measurements in UHV. For other metal oxides, sample preparation at the atomic level is possible in the same way.

### Atomic resolution imaging of insulator metal oxide films with NC-AFM

**Figure 4:**
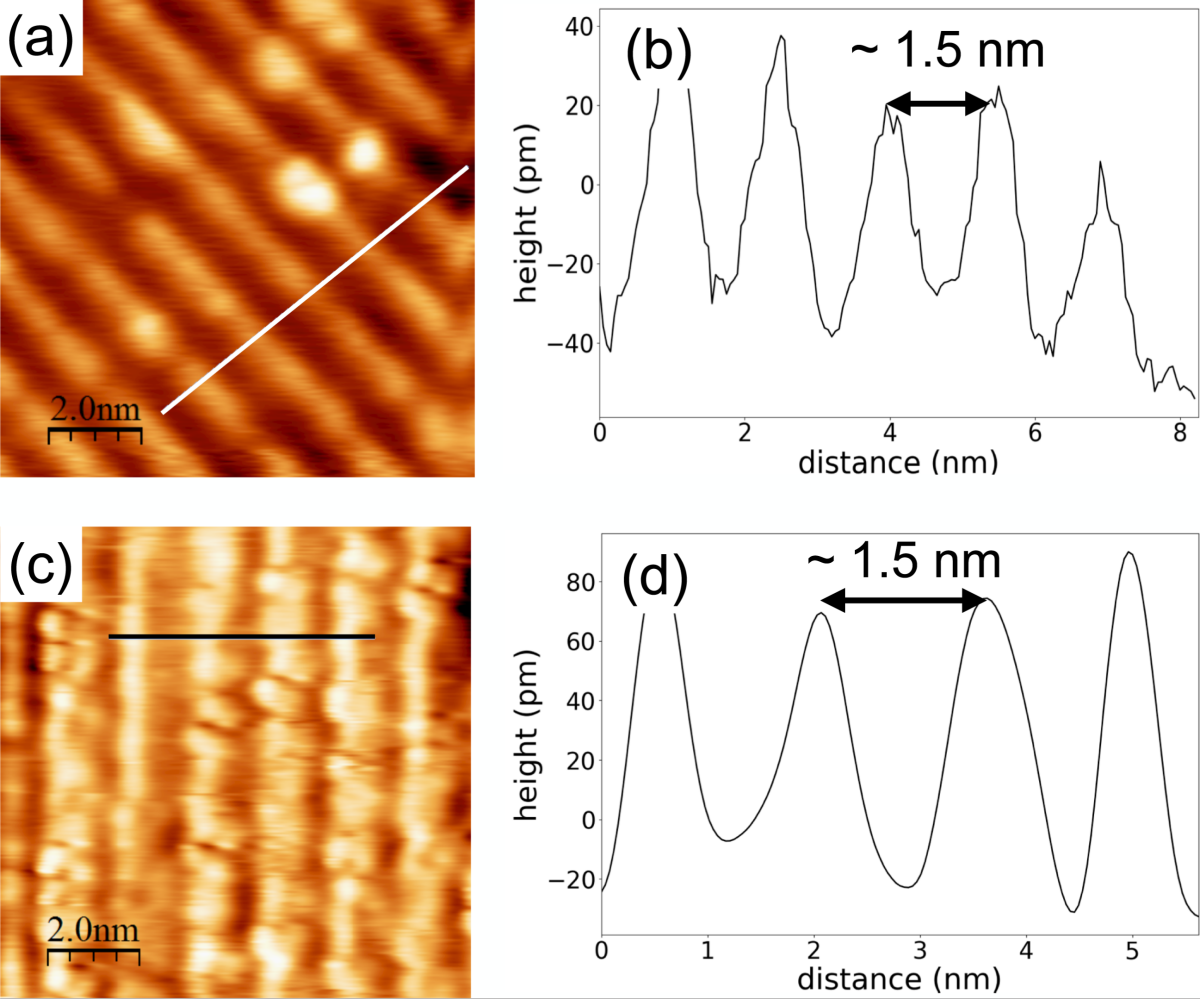
NC-AFM topographic images and line profiles of insulator thin films of (a, b) anatase TiO_2_(001) and (c, d) LaAlO_3_(100). Values of the cantilever resonance frequency, spring constant, oscillation amplitude and frequency shift were *f*_0_ = 167 kHz, *k* = 34.9 N/m, *A* = 14 nm, Δ*f* = −4.2 Hz for (a), and *f*_0_ = 164 Hz, *k* = 32.9 N/m, *A* = 16 nm, Δ*f* = −13 Hz for (b), respectively. The contact potential difference (CPD) was compensated for each image with *V*_s_ = 2.5 V for (a) and *V*_s_ = −0.6 V for (b). The substrates used for PLD were Nb-doped SrTiO_3_(100) (dopant level 0.05 wt %) and non-doped LaAlO_3_(100) for (a) and (c), respectively. Parameters used for thin-film growth of the anatase TiO_2_(001) were *T*_s_ = 800 °C, *P*_O_ ≈ 1 × 10^−4^ Pa, *I* = 0.8 J/cm^2^, *f*_p_ = 2 Hz. For LaAlO_3_(100) thin film growth, the parameters were *T*_s_ = 900 °C, *P*_O_ ≈ 1 × 10^−3^ Pa, *I* = 0.3 J/cm^2^, *f*_p_ = 2 Hz.

Atomic resolution imaging with NC-AFM of insulator thin films of anatase TiO_2_(001) and LaAlO_3_(100) prepared with PLD was carried out. Both materials are important metal oxides, and studies at the atomic level of both materials are still ongoing because of their high importance in materials science. Anatase TiO_2_ is well known as a photocatalyst, but there have been few studies using NC-AFM due to the difficulty in preparing samples [58]. Thin films of LaAlO_3_ epitaxially grown on substrate materials have been studied for correlated electron heterostructures and devices. One of the most important and common uses of epitaxial LaAlO_3_ is its interface with SrTiO_3_ for studies of electrical conductivity [4], superconductivity [59], photoconductivity [60], and magnetoresistance [61]. Figure 4 shows results of atomic-resolution measurements of anatase TiO_2_(001) and LaAlO_3_(100) using the NC-AFM. We determined the PLD conditions according to the procedure mentioned above, described in the caption of Figure 4. LEED measurements were also performed in situ after the NC-AFM measurements. The results showed that the both surfaces had structures with a period of four times the lattice constant (data not shown).

Figure 4a,b shows an atomic resolution image and a line profile of the anatase TiO_2_(001), respectively. From the NC-AFM image and the line profile in Figure 4b, the periodic width of the column was 1.5 nm, which is approximately four times the lattice constant (0.3785 nm). This means that, under these preparation conditions, the anatase TiO_2_(001) surface has been reconstructed to the (1 × 4) or (4 × 1) structure. This result is consistent with the results of LEED and the previous study [23]. Similarly, to the case of anatase TiO_2_(001), the LaAlO_3_(100) surface also has a periodic structure of 1.5 nm. Since the lattice constant of LaAlO_3_(100) is 0.379 nm, the surface was also reconstructed with (1 × 4) or (4 × 1). Details of the NC-AFM image contrast of both surfaces will be described elsewhere.

As shown by the measurement results in Figure 4, by connecting PLD with NC-AFM, we succeeded in atomic-resolution observations of two insulator surfaces that have not been previously measured due to the difficulty of sample preparation. One of the critical points for obtaining atomic resolution images is the sample preparation method described in the first part of section “Results and Discussion”. It has become possible to efficiently find the appropriate conditions for sample preparation with this method. The possibility of studies on surfaces of insulators has thus been greatly expanded by the development of this method. Since it is no longer necessary to repeat the sputtering and annealing that typically takes several days for the preparation of samples, the efficiency of experiments using metal oxides described in this work has dramatically increased. Also, reproducibility of the surface quality can be expected without the sputtering and annealing. Of course, one can perform experiments of the same surface without the combined system. Some insulator thin films such as TiO_2_ made by PLD and left under ambient conditions can be cleaned by an iteration of sputtering and annealing for imaging with atomic resolution. We confirmed that this is the case for the anatase TiO_2_(001) thin film, but not for LaAlO_3_(100). However, even if one can obtain atomic-resolution images, the influence on the surface by, e.g., OH groups, cannot be completely eliminated. Furthermore, the contact potential difference (CPD) of the insulator surface synthesized with our PLD system is at most about 3 V. With our system, it is possible to measure without affecting the charging of the sample surface, which often occurs on insulator surfaces.

## Conclusion

We have developed a combined system of NC-AFM and PLD for atomic-resolution measurements of insulator thin films. The system enabled preparing atomically flat surfaces with high efficiency without the usual iteration of sputtering and annealing. We performed in situ NC-AFM measurements on anatase TiO_2_(001) and LaAlO_3_(100) prepared by PLD and obtained images with atomic resolution. It is expected that atomic-scale studies of the surfaces of insulators that cannot be measured with STM can be carried out using the current system.

## Acknowledgements

We thank Prof. Matsunaga and Prof. Nakamura of Nagoya University, and Prof. Hitosugi and Prof. Shimizu of Tokyo Institute Technology for technical advices on sample preparation and discussions. This work was supported by a Grant-in-Aid for Scientific Research (16K13615, 16H03872, and 25106002) from the Ministry of Education, Culture, Sports, Science and Technology of Japan (MEXT).
